# Femtosecond laser-assisted corneal transplantation with a low-energy, liquid-interface system

**DOI:** 10.1038/s41598-022-11461-9

**Published:** 2022-04-28

**Authors:** Yu-Chi Liu, Fernando Morales-Wong, Moushmi Patil, Sang Beom Han, Nyein C. Lwin, Ericia Pei Wen Teo, Heng Pei Ang, Nur Zah M. Yussof, Jodhbir S. Mehta

**Affiliations:** 1 Tissue Engineering and Cell Therapy Group, Singapore Eye Research Institute, The Academia, 20 College Road, Discovery Tower, Level 6, Singapore, 169856 Singapore; 2 Cornea and Refractive Surgery Group, Singapore Eye Research Institute, Singapore, Singapore; 3 Cornea and External Eye Diseases, Singapore National Eye Centre, Singapore, Singapore; 4 Duke-NUS Graduate Medical School, Ophthalmology Academic Clinical Program, Singapore, Singapore; 5 Faculty of Medicine, University Hospital “Dr Jose Eleuterio Gonzalez”, Autonomous University of Nuevo Leon, San Nicolás de los Garza, Mexico; 6 Department of Ophthalmology, Kangwon National University School of Medicine, Kangwon National University Hospital, Chuncheon-si, Republic of Korea

**Keywords:** Translational research, Surgery

## Abstract

Femtosecond laser-assisted keratoplasty has been proposed as a treatment option for corneal transplantation. In this study, we investigated and compared the outcomes of Ziemer Z8 femtosecond laser (FSL)-assisted penetrating keratoplasty (PK) using a liquid interface versus flat interface. Thirty fresh porcine eyes underwent FSL-assisted PK with the Z8 using different levels of energies (30%, 90% or 150%) and different interfaces (liquid or flat). The real-time intraocular pressure (IOP) changes, incision geometry, corneal endothelial damage, as well as the accuracy of laser cutting and tissue reaction, were performed and compared. We found that the overall average IOP at all laser trephination stages was significantly higher with the flat interface, regardless of the energy used (68.9 ± 15.0 mmHg versus 46.1 ± 16.6 mmHg; *P* < 0.001). The overall mean laser-cut angle was 86.2º ± 6.5º and 88.2º ± 1.0º, for the liquid and flat platform respectively, indicating minimal deviation from the programmed angle of 90º. When high energy (150%) was used, the endothelial denuded area was significantly greater with the flat interface than with liquid interface (386.1 ± 53.6 mm^2^ versus 139.0 ± 10.4 mm^2^ *P* = 0.02). The FSL cutting did not cause obvious tissue reaction alongside the laser cut on histological evaluation. The results indicated a liquid interface is the preferable choice in FSL-assisted corneal transplantation.

## Introduction

Corneal transplantation, including full-thickness penetrating keratoplasty (PK) and selective keratoplasty techniques, such as deep anterior lamellar keratoplasty (DALK), Descemet stripping automated endothelial keratoplasty (DSAEK), and Descemet membrane endothelial keratoplasty (DMEK), remains the main method for the treatment of irreversible corneal diseases. The total number of corneal transplantation performed in the USA increased from 33,260 procedures in 2000, to 51,336 procedures in 2019^1^. In the USA in 2020, 26,095 PK and DALK procedures were performed, accounting for 39.4% of the total number of corneal grafts^1,2^. However, limitations of PK include the higher rates of graft rejection, prolonged visual rehabilitation and high residual astigmatism^3,4^. Hence, it is necessary to continue refining new technology and instrumentation, to potentially allow for better visual and refractive results.

Femtosecond laser (FSL) has been shown to create precise corneal flaps^5,6^, conjunctival grafts^7,8^, as well as lens capsulotomy^9^, with minimal collateral tissue damage^10^. They have been shown to accurately trephine the host and donor corneas for PK, DALK and DSAEK^11–15^. Among FSL-assisted keratoplasties, FSL-assisted PK has been investigated the most. Previous studies have shown good visual results and low degrees of postoperative astigmatism^15–18^. Compared with manual trephination, FSL trephination has also been shown to offer faster visual recovery, due to early removal of sutures and less endothelial cell damage^14,19^. In addition, FSLs allow a variety of customized trephination patterns with reproducible cuts, improving donor-host alignment, donor wound healing, as well as reducing wound leakage, by maximizing the contact area between the donor and receipt with the alternation of cutting angulation^14^. This highlights the importance of accurate incisional geometry during trephination.

Several FSL platforms have been used for corneal trephination for PK^18,20–22^, and they can be classified according to their contact with the cornea as applanating or non-applanating interfaces. A flat-interface system, such as the IntraLase femtosecond laser (Abbott Medical Optics, Santa Ana, USA), may cause corneal deformation and folds in Descemet membrane when applanating the laser head onto the corneas, especially for thin and less rigid corneas, such as keratoconic corneas. Curved laser interfaces, such as with the VisuMax (Carl Zeiss Meditec, Jena, Germany) and Victus (Bausch and Lomb, USA) femtosecond laser systems, have also been introduced in an effort to reduce stress on the corneal tissue. However, curved interfaces have different radii of curvature from receipt corneas and thus will still cause corneal deformation during trephination. Both interfaces will also cause raised intraocular pressure (IOP) following applanation^23^. A newly introduced alternative option is a non-applanation system. This is achieved with a liquid interface that allows the natural curvature of the cornea to maintain its shape, avoids mechanical compression, minimizes the eyeball horizontal torsion as well as vertical tilt, and prevents shearing forces during the trephination^24^. The Femto LDV Z8 (Ziemer Ophthalmic Systems AG, Port, Switzerland) is such a platform. A recent meta-analysis showed that the majority of femtosecond laser-assisted PK have been performed with the Intralase system, followed by the Visumax system^21^.

FSL assisted keratoplasty consists of several steps to the procedure. The first step is laser docking, and this allows the surgeon to accurately place the laser centered on the cornea. At the suction activation step (following docking), there is a rise in IOP which can be detrimental in patients with prior optic nerve damage or previous retinal detachment surgery^25^. It also negatively impacts the highly IOP-sensitive corneal endothelium. The Z8 system, like its predecessor, Femto LDV Z6 system, delivers energy pulses in nanojoule levels per spot^26^. The Z6 model facilitates corneal applications using a flat interface, while the Z8 model has the option of liquid interface available for non-applanating keratoplasties. The Z8 model is also equipped with high resolution anterior segment optical coherence tomography (ASOCT) imaging system that can be used intraoperatively during corneal surgery. We have previously compared the real-time IOP changes with the Z8 liquid applanation versus the Z6 flat applanation system for cataract surgery^27^. We found that the IOP was significantly lower with the Z8 liquid interface during the fragmentation/capsulotomy stage compared to the Z6 flat interface during the flap creation (72.5 ± 24.2 mmHg versus 201.9 ± 18.5 mmHg respectively)^27^.

In this study, we aimed to compare the Z8 FSL-assisted keratoplasty with liquid interface versus flat interface, with respect to real-time IOP changes, incision geometry, endothelial cell damage and histological tissue reaction, using a porcine model.

## Results

### IOP changes during trephination

**Figure 1:**
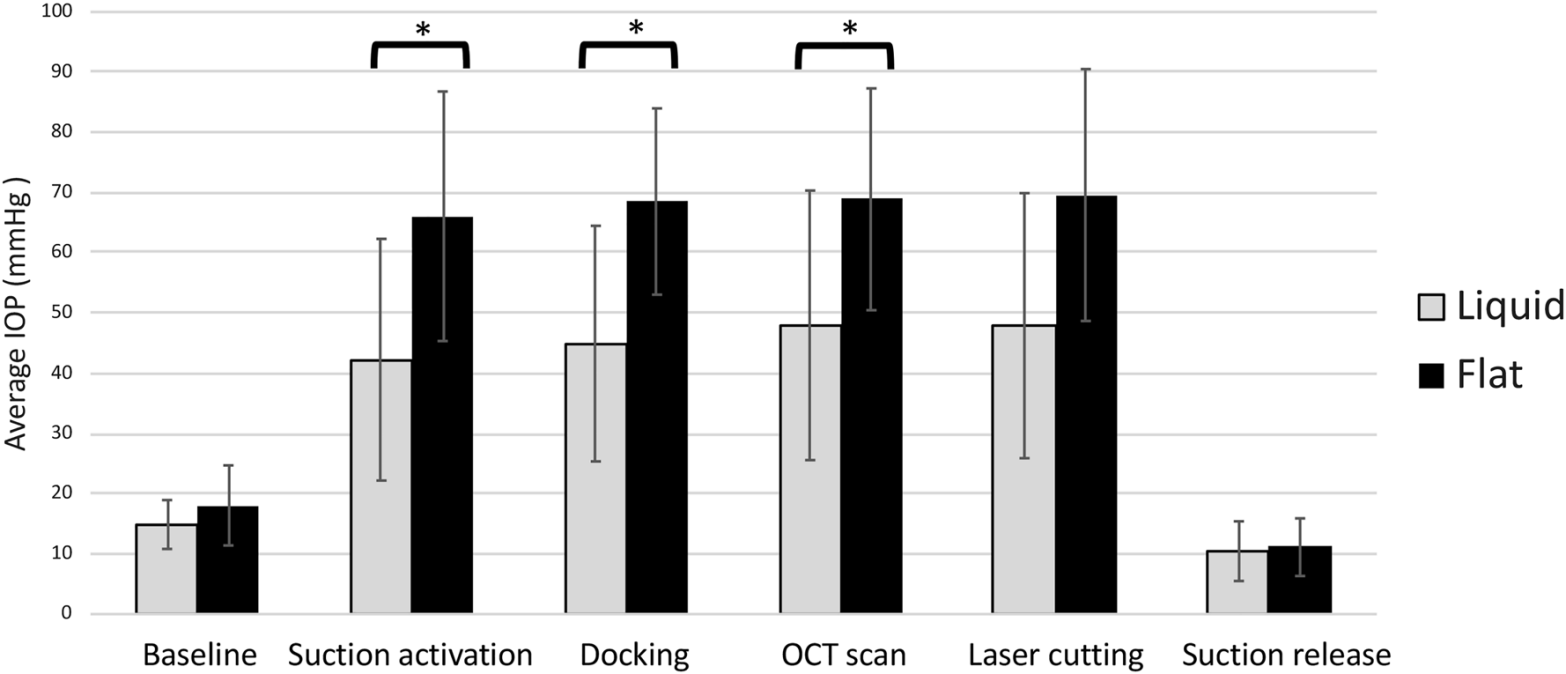
Mean IOP measurements during different phases of the laser trephination with both interfaces. * indicates *P* < 0.05.

Overall, the IOP increased when the suction was applied to all the eyes. Baseline IOP was 18.0 ± 6.6 mmHg for flat interface and 14.9 ± 4.0 mmHg for liquid interface (*P* = 0.17). The average IOP during all the stages in the laser trephination was significantly higher in the flat interface groups regardless of the energy used (46.1 ± 16.6 mmHg and 68.9 ± 15.0 mmHg for the liquid and flat interface groups, respectively; *P* < 0.001). IOP was significantly higher with the flat interface compared with liquid interface in the following stages: suction activation (66.0 ± 20.7 mmHg versus 43.3 ± 20.0 mmHg; *P* = 0.02), docking (68.5 ± 15.5 mmHg versus 45.1 ± 19.5 mmHg; *P* = 0.01), OCT scan (68.8 ± 18.4 mmHg versus 49.5 ± 22.3 mmHg; *P* = 0.04), and laser cutting (69.6 ± 20.9 mmHg versus 48.5 ± 21.9 mmHg; *P* = 0.05). Figure 1 shows the mean IOP changes during the trephination with both interfaces.

### Endothelial cell damage

**Figure 2:**
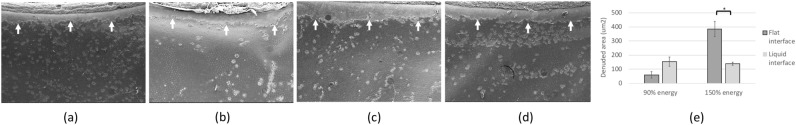
Representative electronic microscopy images showing the endothelial denuded area (arrows) with different interfaces and different levels of energy qualitatively and quantitatively: flat interface with 90% energy (**a**), liquid interface with 90% energy (**b**), flat interface with 150% energy (**c**), and liquid interface with 150% energy (**d**). Bar graph showing the comparisons of the measured denuded areas (**e**). * indicates *P* < 0.05.

The denuded areas adjacent to the trephination incision on SEM images were measured. With 90% energy, the FSL created a clean cut with a minimal and similar extent of collateral endothelial cell damage in the liquid and flat interface groups. The mean denuded area was 60.0 ± 22.3 mm^2^ and 154.0 ± 32.1 mm^2^, for the liquid and flat interface, respectively (*P* = 0.08). In contrast, damage to endothelial cells was significantly greater with the flat interface when using 150% energy (386.1 ± 53.6 mm^2^) compared to the liquid interface (139.0 ± 10.4 mm^2^; *P* = 0.02) (Fig. 2). The laser cuts in the 30% energy group were incomplete due to the low energy used, and the separation of the tissue bridges was done with scissors manually, which introduced significant endothelial damage and did not reflect the real laser effect on the endothelium. Hence, the results for the 30% groups were not included in the statistical analysis.

### Laser incision geometry

**Figure 3:**
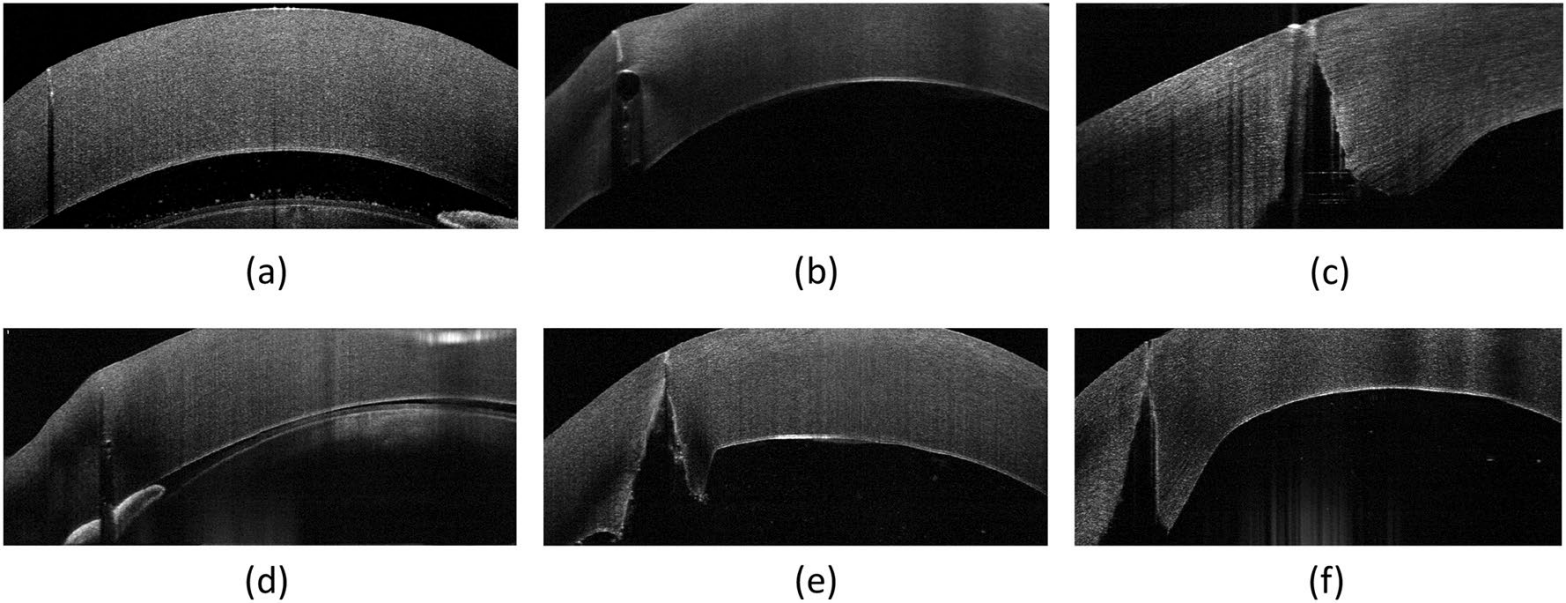
Representative ASOCT images showing the trephine incisions after FSL trephination with different interfaces and different levels of energy. Flat interface with 30% energy (**a**), flat interface with 90% energy (**b**), flat interface with 150% energy (**c**), liquid interface with 30% energy (**d**), liquid interface with 90% energy (**e**), and liquid interface with 150% energy (**f**).

On ASOCT images, the laser cutting path was visible, and no abnormal hyperreflectivity resulting from the laser photo-disruption was seen in the stroma in all the corneas (Fig. 3). Overall, the mean laser cut angle was 86.1º ± 6.4º and 88.1º ± 1.0º, and the mean uncut angle was 89.4º ± 0.9º and 89.2º ± 1.5º, for the liquid and flat platform (*P* = 0.52, *P* = 0.41, respectively), indicating a small deviation (0.6 to 4.3%) from the programmed angle of 90º. The measured cut angle and uncut angle for both interfaces at different energy levels are shown in Table 1.

**Table 1:** The cut and uncut angle measured for both interfaces at different levels of energy.

	Energy level	Cut angle (º)	Uncut angle (º)
Flat interface	30%	88.8 ± 0.3	89.0 ± 0.6
	90%	88.3 ± 1.4	88.3 ± 1.5
	150%	88.3 ± 0.7	89.7 ± 1.6
	*P* value*	0.10	0.16
Liquid interface	30%	89.1 ± 0.1	87.5 ± 0.7
	90%	83.1 ± 8.7	89.8 ± 0.5
	150%	88.7 ± 1.0	89.6 ± 0.8
	*P* value*	0.10	0.07
Comparisons between flat versus liquid interfaces with 30% energy	*P* value	0.43	0.12
Comparisons between flat versus liquid interfaces with 90% energy	*P* value	0.79	0.09
Comparisons between flat versus liquid interfaces with 150% energy	*P* value	0.18	0.33

*Comparisons among three energy groups.

### Histology

**Figure 4:**
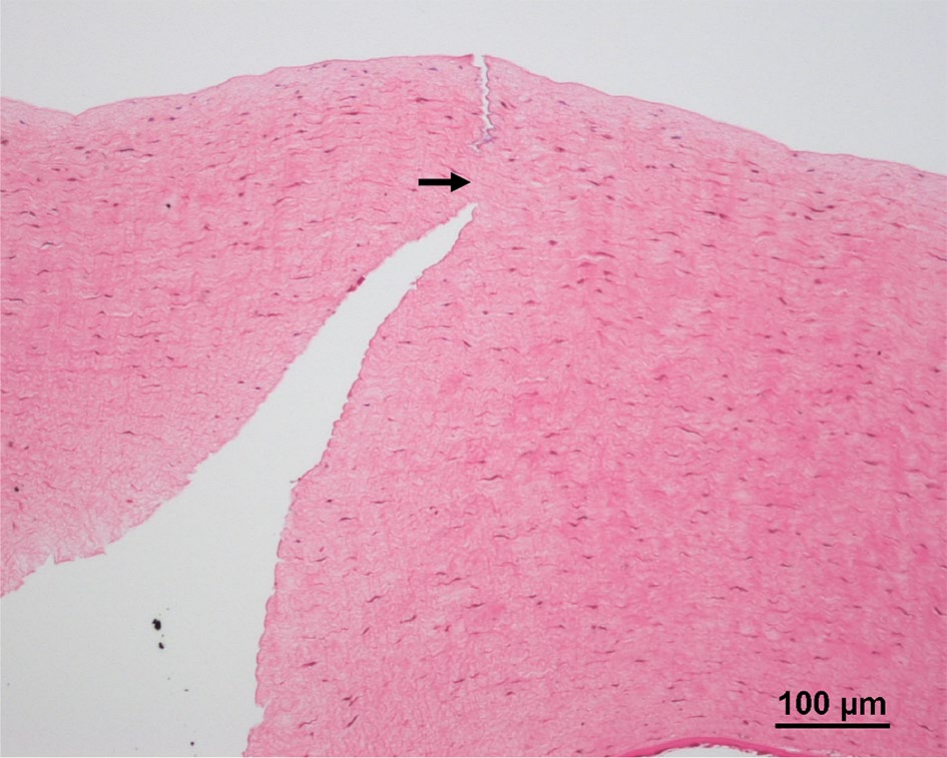
Representative histological section. On histological evaluation, FSL did not cause obvious coagulative necrosis, inflammation, or thermal burn along the laser path irrespective of the energy used. Arrow indicates the uncut area.

The FSL did not cause obvious coagulative necrosis, inflammatory reaction and thermal burn in the stromal tissue surrounding the laser cut irrespective of the energy used. The mean uncut length and corneal thickness measured were 51.9 ± 11.2 μm and 540.2 ± 40.7 μm with flat interfaces (i.e. the uncut length = 9.6%), and were 53.0 ± 16.5 μm and 445.6 ± 66.7 μm with liquid interfaces (i.e. the uncut length = 11.9%). These results indicate the accuracy of laser cutting as the uncut length was set at 10% in all the cases (Fig. 4).

## Discussion

In the present study, we have demonstrated that corneal trephination for PK with a FSL using a liquid interface had significantly less IOP rise and better stability of IOP than a flat interface. The laser cut with the liquid interface was accurate with minimal endothelial cell damage, and no obvious stromal tissue reaction was observed on histology. The effects on the endothelium with a liquid interface, compared to a flat interface, were significantly less when high laser energy was used. The results provide evidence that a liquid interface is the preferable choice than a flat interface in corneal transplantation in a porcine model.

This study reported the real-time IOP changes during trephination with two different interfaces and showed significant differences. It is advantageous to maintain intraoperative IOP as constant as possible, to avoid the risk of retinal vascular occlusion, retinal detachment, patient discomfort, glaucoma progression or any other high IOP-related complications^25,28^. The better IOP stability associated with a liquid interface may therefore improve the safety profiles in patients vulnerable to high IOP or IOP fluctuations when employing FSL for PK. Moreover, it is known that IOP affects the curvature of the cornea, and hence maintaining a stable pressure during trephination is also important to keep the correct plane and to avoid irregular cuts of tissue.

We have previously demonstrated that the IOP remained constant during different cutting phases with a curved applanating interface, preventing tissue herniation. On the contrary, manual trephination caused IOP spikes during these phases^19^. In the present study, the liquid interface had significantly lower IOP in all the phases of the laser trephination. The differences in the IOP resulted from the mechanical compression on the cornea, in addition to any pressure caused by the surgeon inadvertently pressing down when using a flat interface^19^. Previous studies on flat applanation systems have also shown increased IOP during the vacuum phase in FSL-assisted flap creation, with the mean IOP exceeding 90 mmHg, compared to 48 to 65 mmHg with curved applanation interfaces^19,29–31^. Ebner et al. showed that during the suction period, with a vacuum of 350 and 420 mbar, the mean IOP was 45.2 ± 4.3 mmHg and 52.0 ± 6.4 mmHg, respectively, with the LDV Z8 liquid interface for cataract surgery^32^. This was in agreement with our results in which the mean IOP during all suction phases was 46.1 ± 16.6 mmHg with the same liquid interface. Similarly, Choi et al. reported that the mean IOP was in a range from 96.6 to 138.4 mmHg with the Intralase applanation interface, in comparison with 48.5 mmHg with the Femto LDV Z8 liquid system, in FSL-assisted keratoplasty^33^.

A good trephination geometry helps to obtain good alignment in the graft-host junction, resulting in better wound stability, less wound leakage and less induced astigmatism^34^. Conventional manual hand-held trephines tend to undercut the tissue, producing a misalignment known as “vertical tilt”, in which the diameter from the epithelial side is smaller than the endothelial side in the host cornea. This can be due to several factors such as intraoperative IOP fluctuations and surgeons’ excessive compression. Reproducible trephination with congruent borders that fit and align among the host and the donor cornea can be achieved with FSL^34^. In this study, the mean angle achieved in both interface groups was close to the programmed angle of 90º, suggesting an accurate trephination. A 10% uncut tissue thickness, as suggested according to the Ziemer surgical manual^35^, was programmed to avoid full-thickness trephination and anterior chamber collapse. A 10% uncut tissue would be enough to prevent complete collapse of the anterior chamber while not allowing too much tissue that need to be manually cut which is associated more tissue manipulation, and this uncut angle was also close to the programmed angle of 90º. Contrasting to the flat interface, where applanation distorts the cornea, the non-applanating liquid interface has the advantage of preserving the natural corneal curvature, and the absence of corneal deformation helps to have more congruent incision edges^24^. This is especially useful in patients with thin or less rigid corneas such as keratoconus in which applanating the cornea causes non-circular openings due to compression and distortion^36^. The Catalys femtosecond laser system (ForTec Medical, Ohio, USA) also has a liquid non-applanating interface available for cataract surgery, but no report was published for keratoplasty to our knowledge^37^.

It is advantageous to reduce corneal endothelial cell damage during trephination. Moreover, increased laser energy during the trephination would be required when there is the presence of corneal edema or corneal opacity, which is the main indication for PK. We found that the flat interface group had greater endothelial denuded areas than the liquid interface group in both 90% and 150% energy settings, and the difference was even more significant when high energy was used (386.1 ± 53.6 mm^2^ versus 139.0 ± 10.4 mm^2^; *P* = 0.02). The difference may be because the higher energy makes cells more susceptible to mechanical stress^38^. This would also highlight the advantages of using a liquid interface in FSL-assisted PK for edematous corneas. In addition, impacts on corneal endothelium with FSL-assisted and manual trephination have been studied. It was reported that the endothelial cell damage was three to four times more when the trephination was performed with conventional manual trephine compared to FSL^19,39^. Similar results have been shown in clinical studies. Bahar et al. found significantly less endothelial cell count 1 year after PK in patients whose keratoplasties were done with manual trephines, compared to those patients who underwent FSL-assisted keratoplasties^40^. Another study also showed more endothelial cell loss in conventional manual PK compared to FSL-assisted keratoplasty with a flat applanation interface^41^.

Fresh cadaveric pig eyes within six hours of retrieval were used for the experiments, and the corneas were still thicker than human corneas due to inevitable corneal edema. Therefore, the IOP measured may have been over-estimated. However, we focused on the comparisons across the experimental groups, and each group had the same experimental setup and characteristics of porcine eyes. The corneal status of porcine eyes would also simulate clinical corneal edema. Moreover, although porcine corneas could not completely simulate human corneas, a porcine model has been extensively used in the field of FSL corneal surgery research^42,43^. Some evaluation in this study, such as histological evaluation and endothelial denuded area assessed by scanning electron microscopy, could not be performed in patients’ eyes clinically and had to be carried out with a porcine model.

In conclusion, we present a comprehensive study in which we compared liquid and flat interfaces in FSL-assisted keratoplasty. Both of the interfaces offered intraoperative adjustment of the trephination size and thickness guided by intraoperative OCT. The liquid interface was associated with lower IOP and less extent of IOP fluctuations during the procedure compared with the flat interface, offering the advantage of fewer IOP-related complications. The laser cutting was accurate, allowing for better graft-host apposition. The non-applanation liquid interface maintained the anatomical curvature of the cornea and caused less endothelial cell damage, particularly when a high-energy setting was required. Our study provides favorable evidence supporting future clinical applications in not only PK but also lamellar keratoplasty for a liquid interface, and future clinical trials are required to attest the results obtained from this porcine study and to evaluate long-term clinical results. Surgeons with access to a liquid interface-based FSL in their practice may consider the use of this device for trephination during corneal graft surgery.

## Methods

### Experimental groups, laser procedure, and real-time IOP measurement

Thirty fresh porcine eyes were used. These eyes were within six hours of retrieval from a local abattoir and submerged in Optisol (Bausch & Lomb, Inc. USA) to prevent corneal swelling from enucleation. The eyes were allocated into the following experimental groups: 150% energy with liquid interface (n = 6), 150% energy with flat interface (n = 6), 90% energy with liquid interface (n = 6), 90% energy with flat interface (n = 6), 30% energy with liquid interface (n = 3), and 30% energy with flat interface (n = 3).

After corneal epithelial debridement, the eyes were mounted on a pressurized stand, and a 30-gauge cannula connected to an IOP catheter transducer was inserted into the anterior chamber, posterior to the limbus. The LabChart 6 (ADI Instruments, Dunedin, New Zealand) transducer was used according to the manufacturer’s instructions, and calibration was performed before starting each trephination. Baseline IOP was also measured three times with a Tonopen (Reichert-Jung, Depew, USA), and the average was taken as a reference of measurement of intracameral IOP. Real-time IOP was measured for the following steps with continuous recording of IOP: baseline, suction activation, docking of the laser handpiece, intraoperative ASOCT scan, laser cutting of the tissue, and suction release.

**Figure 5:**
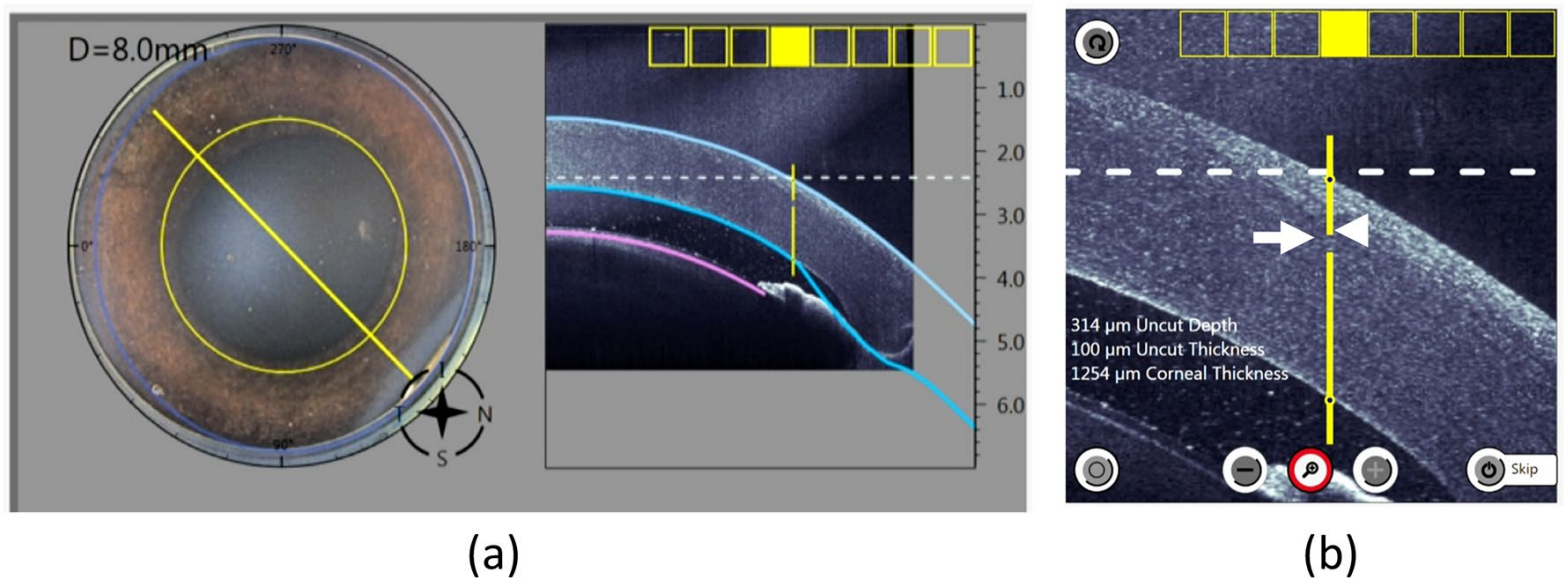
ASOCT scans were performed intraoperatively to mark the centration of the trephination and to adjust the thickness of the cut. Superior view showing the preselected diameter for the trephination (**a**). ASOCT shows the trephination line with the area where the uncut thickness and depth were (**b**; arrow and arrowhead, respectively).

**Figure 6:**
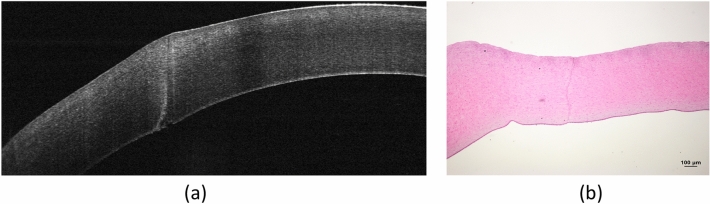
ASOCT and a representative histological section showing laser trephination with 30% energy. Both ASOCT images (**a**) and histological section (**b**) showed that the trephination was not cut through.

For the FSL-assisted PK procedure, each procedure was carried out with standard clinical settings. For the flat interface groups, an 8.5 mm flat-applanating handpiece was docked onto the eye with the centration over the limbus. For the liquid interface groups, the suction ring was first applied to the eye, and it was then filled with 3–5 mL balanced salt solution to create a fluid–corneal interface, without the necessity of applanation of the cornea. ASOCT scans were performed with the in-built intraoperative ASOCT, to mark the centration of the trephination and to adjust the laser cutting parameters (Fig. 5). The laser parameters were: side cut angle of 90º, trephination diameter of 8.0 mm, anterior uncut depth of 25%, uncut area thickness of 10%, cut speed of 50 mm/s, repetition rate at 2 MHz, pulse duration at 250 fs, and energy of 30% (lowest laser energy setting), 90% (recommended laser setting), and 150% (close to the highest laser energy setting which is 160%)^35^. Trephination with 30% energy was performed on three eyes only for each interface as the laser did not cut through the cornea due to low energy used, and trephination was subsequently completed with corneal scissors (Fig. 6).

### Evaluation of laser incision geometry

**Figure 7:**
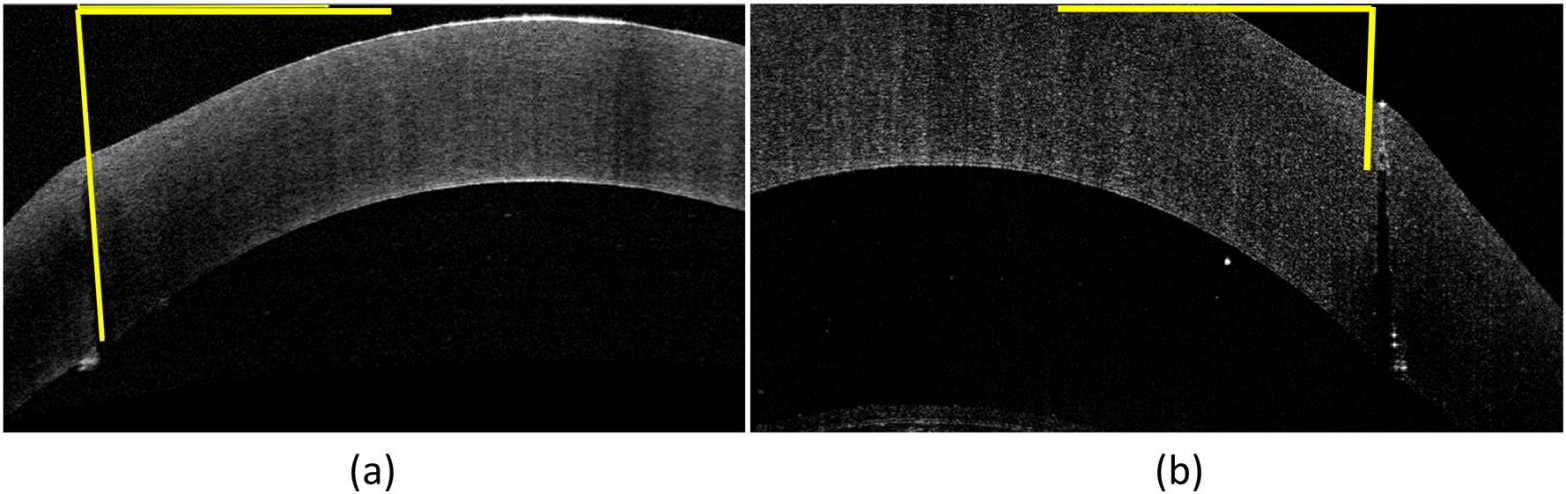
Post laser-cutting ASOCT images showing how the angles were measured. For the measurement of the laser cut angle, the first line was drawn in the horizontal plane of the image, and the second line was drawn parallel to the cut stroma. The angle formed by the two lines was the cut angle (**a**). Similarly, the angle formed by the line of the uncut stroma and horizontal plane was the laser uncut angle (**b**).

The cutting geometry was immediately evaluated by ASOCT (RTVue; Optovue, Inc., USA). For each cornea, a total of 4 high-resolution corneal cross-sectional scans (8 mm scan length, single scan mode, approximately 45 axis apart) were obtained. As stated above, an uncut area was left in all the corneas to prevent perforation. The angle formed between the cut stroma and the uncut stroma was measured by one observer using ImageJ (NIH, Bethesda, USA) as follows: a line was drawn in the horizontal plane of the ASOCT scan, and the other line was drawn parallel to the stroma cut in the donor site. The angle formed within the intersection of both lines was measured, and the average of four measurements was used (Fig. 7a). The same step was performed using the uncut stroma as reference to get the uncut angle (Fig. 7b).

### Evaluation of endothelial denuded area

The endothelial denuded area along the laser trephination was evaluated with scanning electron microscopy (SEM). Corneas were fixed in neutral buffered 2% glutaraldehyde (Electron Microscopy Sciences, Hatfield, USA) at 4 °C for 24 h. After rinse with 1 × PBS, they were cut into halve and post-fixed in aqueous solution of 1% osmium tetroxide at room temperature for 30 min. The samples were then dehydrated under an increasing alcohol gradient: 25% ethanol for 5 min, 50% ethanol for 5 min, 75% ethanol for 5 min, 95% ethanol for 5 min, 3 × 100% ethanol for 10 min each, followed by critical point drying (BALTEC, Balzer, Liechtenstein). Dehydrated samples were then mounted onto a metal stub using carbon adhesive tabs. Samples were sputter-coated with a 25-nm layer of gold–palladium alloy (BALTEC), and examined under a scanning electron microscope (Quanta 650FEG; FEI, Hillsboro, OR). The denuded endothelial area, alongside the laser path for each cornea sample, was measured using ImageJ (National Institutes of Health, Bethesda, MD). The denuded area was defined as regions absent of endothelial cells from the laser cut.

### Histology evaluation

The histological evaluation was performed as previously described^44,45^. In brief, the tissue samples were fixed in neutral 4% buffered paraformaldehyde, and then were dehydrated, cleared, and embedded in paraffin, before being cut in 7 μm sections. Hematoxylin and eosin were used to stain the sections, and the images of sections were examined using a light microscope (Axioplan, Carl Zeiss MicroImaging, USA) under bright field mode. The corneal thickness and the uncut length on histological sections were measured using ImageJ software.

### Statistical analysis

The primary outcome of the present study was the average IOP during all the stages in the laser trephination. The required sample size was calculated based on the pilot data on the average IOP, which was 49.1 ± 14.5 mmHg and 69.2 ± 13.7 mmHg for the flat and liquid interface groups, respectively (n = 3 for each group). A sample size of 9 eyes per arm, with a power of ≥ 80% and at a 5% significance, was therefore sufficient to detect the difference between the 2 groups. All data were expressed as mean ± standard deviation. Mann–Whitney U test was used to compare the data between the flat and liquid interface groups, and Kruskal–Wallis test to analyze the data across the 30%, 90%, and 150% energy groups. A P value of ≤ 0.05 was considered statistically significant.
